# Expressions of Tight Junction Proteins Occludin and Claudin-1 Are under the Circadian Control in the Mouse Large Intestine: Implications in Intestinal Permeability and Susceptibility to Colitis

**DOI:** 10.1371/journal.pone.0098016

**Published:** 2014-05-20

**Authors:** Kyoko Oh-oka, Hiroshi Kono, Kayoko Ishimaru, Kunio Miyake, Takeo Kubota, Hideoki Ogawa, Ko Okumura, Shigenobu Shibata, Atsuhito Nakao

**Affiliations:** 1 Department of Immunology, University of Yamanashi Faculty of Medicine, Yamanashi, Japan; 2 The First Department of Surgery, University of Yamanashi Faculty of Medicine, Yamanashi, Japan; 3 Department of Epigenetic Medicine, University of Yamanashi Faculty of Medicine, Yamanashi, Japan; 4 Atopy Research Center, Juntendo University School of Medicine, Tokyo, Japan; 5 Department of Physiology and Pharmacology, School of Advanced Science and Engineering, Waseda University, Tokyo, Japan; University of Chicago, United States of America

## Abstract

**Background & Aims:**

The circadian clock drives daily rhythms in behavior and physiology. A recent study suggests that intestinal permeability is also under control of the circadian clock. However, the precise mechanisms remain largely unknown. Because intestinal permeability depends on tight junction (TJ) that regulates the epithelial paracellular pathway, this study investigated whether the circadian clock regulates the expression levels of TJ proteins in the intestine.

**Methods:**

The expression levels of TJ proteins in the large intestinal epithelium and colonic permeability were analyzed every 4, 6, or 12 hours between wild-type mice and mice with a mutation of a key clock gene Period2 (Per2; mPer2^m/m^). In addition, the susceptibility to dextran sodium sulfate (DSS)-induced colitis was compared between wild-type mice and mPer2^m/m^ mice.

**Results:**

The mRNA and protein expression levels of Occludin and Claudin-1 exhibited daily variations in the colonic epithelium in wild-type mice, whereas they were constitutively high in mPer2^m/m^ mice. Colonic permeability in wild-type mice exhibited daily variations, which was inversely associated with the expression levels of Occludin and Claudin-1 proteins, whereas it was constitutively low in mPer2^m/m^ mice. mPer2^m/m^ mice were more resistant to the colonic injury induced by DSS than wild-type mice.

**Conclusions:**

Occludin and Claudin-1 expressions in the large intestine are under the circadian control, which is associated with temporal regulation of colonic permeability and also susceptibility to colitis.

## Introduction

The intestinal epithelium forms the boundary between the body and external environment and provides a selective permeable barrier that limits the permeation of luminal harmful molecules, while allowing the appropriate absorption of nutrients and water. This selective permeability in the intestine depends on specialized structures composed of tight junctions (TJs) [1]–[3]. TJs are multiple protein complexes consisting of Occludin, Zonula occludens (Zo), Claudins, and junctional adhesion molecules (JAM), located at the apical ends of the lateral membranes of epithelial cells [2], [3].

The circadian clock drives daily rhythms in physiology and behavior that enable organisms to keep track of the time of day [4]–[6]. Mammalian circadian clock consists of the central oscillator located in the suprachiasmatic nucleus (SCN) of the hypothalamus and peripheral oscillators present in nearly all cell types [4]–[6]. The central SCN clock is set by light and synchronizes the peripheral clocks via neural and endocrine pathways. At the cellular levels, the circadian clock is based on autonomous interlocking transcriptional-translational feedback loops consisting of several “clock genes”. Briefly, the PAS domain helix-loop-helix proteins Clock and Bmal1 bind as heterodimers to regulatory elements of Cryptochrome (Cry) and Period (Per) genes via E-box and stimulate the transcription of these genes. Once the repressor proteins Cry and Per have reached a critical concentration, they attenuate the activity of Clock-Bmal1 heterodimers and thereby repress the transcription of their own genes. These core molecular clocks also control expression of clock-controlled genes (CCGs) via direct or indirect transcriptional activation through clock-controlled transcriptional regulators.

Previous studies suggest that the circadian clock is present in intestinal epithelial cells and plays an important role in several functions of intestinal epithelial cells, such as retaining homeostasis, cell division and lipid absorption [7]–[9]. A recent study suggested that intestinal permeability was also regulated by the circadian clock [10]. However, the precise mechanisms how the circadian clock affects intestinal permeability remain largely unknown. The primary aim of this study is to investigate whether the expression levels of TJ proteins that play an important role in intestinal permeability are under control of the circadian clock.

## Methods

### Mice

Female 5- to 6-week-old ICR mice purchased from Japan SLC (Tokyo, Japan), ICR mPer2^m/m^ and Clock^Δ19/Δ19^ mice were bred under specific pathogen-free conditions. All mice were housed in 12-hours light/12-hours dark conditions (L/D cycles; the light was turned on at 6∶00 AM, Zeitgeber time [ZT] 0, and the light was turned off at 6∶00 PM, ZT12) with ad libitum access to food and water, for at least 2 weeks. All animal experiments were approved by the institutional review board of the University of Yamanashi and the Committee for Animal Experimentation of the School of Science and Engineering at Waseda University.

### Quantitative Real-time PCR (Q-PCR)

A quantitative real-time PCR analysis using cDNA from the mouse tissue specimens was performed using the AB7300 real-time PCR system (Applied Biosystems, Foster City, CA, USA) according to the manufacturer’s instructions, using primers and probes for mouse Occludin, Zonula occludens (Zo)-1, Claudin-1, 2, 3, 4, 7, Junctional adhesion molecule (JAM), Myeloperoxidase (Mpo), Heme oxygenase (Ho)-1, Cyclooxygenase (Cox)-2, Prostaglandin E synthetase (Pges)-1, Per1, 2, 3, Clock, Bmal1, Cry1, IL-1β, TNF-α and Glyceraldehyde-3-phosphate dehydrogenase (GAPDH; Applied Biosystems) as previously described [11]. The ratio of each gene to that of GAPDH was calculated, and the relative expression levels are shown.

### Isolation of Intestinal Epithelial Cells (IECs)

IECs were isolated as described previously [12]. Briefly, an inverted large intestine of mice was cut into four segments and the segments were transferred to a 50-ml tube containing 10 ml RPMI1640 medium/5% FCS/25mM Hepes. The tube was shaken at 37°C at 100 rpm for 45 min. After incubation, vortex the cell solution vigorously for 20s and pass through a 70 µm cell strainer. Subsequently, the cells were suspended in 30% (wt/vol) Percoll and centrifuged at 20°C at 400×g for 20 min. After centrifugation, cells at the top of the 30% Percoll were enriched with IECs.

### Western Blot Analysis

IECs were lysed in buffer containing 0.1 M Tris-HCl (pH 7.5)/2% SDS/10% glycerol/5% 2-mercaptoethanol, boiled at 95°C for 5 min and centrifuged at 15000 rpm for 5 min and analyzed by reducing SDS-PAGE. Immunoblots were performed using antibodies against Claudin-1 (Abcam, Cambridge, UK, ab15098, 1∶100), Occludin (Applied Biosystems, 71–1500, 1∶250), Clock (Abcam, ab3517, 1∶200), Bmal1 (Abcam, ab3350, 1∶200) and β-actin (Santa Cruz, Texas, sc-1615, 1∶200). Quantitative analysis of Western blotting was done by using the Scion Image Software package and relative intensities of the target proteins to β-actin were shown.

### Chromatin Immunoprecipitation (CHiP) Assay

CHiP was performed using SimpleChIP® Enzymatic Chromatin IP Kit (Agarose Beads) (Cell signaling, Tokyo, Japan, #9002). Isolated IECs or cultured cells (HCT116 and Caco-2) were cross-linked in 1% formaldehyde, followed by the CHiP assay according to the manufacturer’s protocol. Rabbit polyclonal antibodies to CLOCK (ab3517) and BMAL1 (ab3350) were from Abcam (Cambridge, UK). DNA was purified and Q-PCR was done using SYBRgreen reagent (QIAGEN, Hilden, Germany). Primers used were as follows: mouse Occludin promoter forward 5′-CTCCCATCCGAGTTTCAGGT-3′ and reverse 5′-GCTGTCGCCTAAGGAAAGAG-3′, mouse Claudin-1 promoter forward 5′-GTTTGCAGAGACCCCATCAC-3′ and reverse 5′-AGAAGCCAGGATGAAACCCA-3′, human Occludin promoter forward 5′-CTCCCATCCGAGTTTCAGGT-3′ and reverse, human Claudin-1 promoter forward 5′-CTCCCCGCCTTAACTTCCTC-3′ and reverse 5′-CAGGAAGGCGAGAATGAAGC-3′, 5′-GGAGTGTAGGTGTGGTGTGT-3′.

### Isolation of Promoter Fragments and Reporter Assay

A human Occludin (–60 to +240) or Claudin-1 (–1010 to +360) promoter fragment containing E-box elements was amplified by PCR from genomic DNA of TIG120 cells and cloned into the pGL3 vector (Promega, Fitchburg, Wisconsin) upstream of firefly luciferase. Primer sequences used were as follows: human Occludin promoter forward 5′-CTCGAGAAAGTGCTGAGTGCCTGGAC-3′ and reverse 5′-AAGCTTTCGGTGACCAATTCACCTGA-3′, human Claudin-1 promoter forward 5′-CTCGAGGGAAACTACAGTCCCAGCGA-3′ and reverse 5′-AAGCTTGATGTTGTCGCCGGCATAGG-3′. These reporter constructs were transfected into HCT-116 or Caco-2 cells cultured in 96-well plates using Fugene6 (Promega) in the presence of pcDNA3, pcDNA3-Clock or pcDNA3-Bmal1 expression plasmid. pcDNA3-Clock and pcDNA3-Bmal1 expression plasmids were kindly gifted by Dr. Fuyuki Sato (Hirosaki University, Aomori, Japan). At 48 hours after transfection, the luciferase activity was measured using the luciferase reporter assay system (TOYO ink, Tokyo, Japan).

### *In vivo* Colon Permeability Assay

Colon permeability to Evans blue was measured *in vivo*. This is a well-validated method for assessing epithelial permeability [13]. In isoflurane-N_2_O anesthesia, spontaneously breathing animals were placed in a supine position on a heating pad and a laparotomy was performed. A small polyethylene tube (G22) was inserted into the proximal colon ascendens (immediately adjacent to the cecum) and secured by a ligature. Via this tube the colon was gently flushed until all stool was rinsed out, and 1 ml of Evans blue 1.5% (Sigma-Aldrich Japan, Tokyo, Japan, E2129) in PBS was instilled into the colon and left in place for 15 min. Then the colon was rinsed with PBS, until the perianal washout was clear. Animals were euthanized, and the colon was rapidly taken out. It was rinsed again with several milliliters of PBS, followed by 1 ml of 6 mM N-acetylcysteine to eliminate dye sticking in the colonic mucus. The colon was opened and rinsed once more with PBS, and its length was recorded. The whole colon was placed in 2 ml N,N-dimethylformamide for 12 h to extract the Evans blue dye. The dye concentration in the supernatant was measured spectrophotometrically at 610 nm and given as extinction per gram colonic tissue.

### *Ex vivo* Colon Permeability Assay

Colon permeability was measured in isolated intestinal segments using FITC-dextran or Horseradish peroxidase (HRP) as described previously [14], [15]. Briefly, 6-cm segments of the large intestine were removed, rinsed with ice-cold saline, everted, filled with 700 µl PBS and ligated at both ends. The filled colon segments were incubated in PBS containing 1 mM FITC-dextran (average mol wt, 4000 Sigma-Aldrich Co. Missouri, USA) or 0.4 mg/ml HRP (type IV, 300 units/mg solid, Sigma-Aldrich Co.). The colon sacs were removed after 45 minutes and the contents (∼500 µL) of each sac was collected carefully using a 1-ml syringe. The amount of FITC-dextran that transverse the colon was quantified by fluorescence plate reader at 521 nm. The HRP activity in the contents of each sac was determined spectrophotometrically based on the rate of oxidation of pyrogallol.

### Induction of Dextran Sodium Sulfate (DSS)-induced Colitis

Colitis was induced by feeding mice 5% DSS dissolved in drinking distilled water (mol wt 5000; Wako Pure Chemical Co., Osaka, Japan) for 7 days, as previously described [16].

### Stool Scores

The stool scores were calculated based on a method by Cooper et al. with some modifications [17]. Briefly, the scoring was based on a maximum score of 4. Stool consistency was graded as: 0 = firm, 1 = loose, 2 = diarrhea. Blood in the stool was also evaluated on a 0- to 2-point scale. 0 = no blood, 1 = occult blood, 2 = gross rectal bleeding.

### Enzyme-Linked Immunosorbent Assay (ELISA)

The concentrations of tumor necrosis factor-α (TNF-α) and interleukin-6 (IL-6) in the mouse colon tissue were determined using the mouse TNF-α and IL-6 enzyme-linked immunosorbent assay (ELISA) kits (R&D Inc, Minneapolis, MN), respectively, according to the manufacturer’s instructions.

### Generation of Bone Marrow Chimeric Mice

Recipient wild-type mice or mPer2^m/m^ mice were lethally irradiated with 1050 rads using a Co irradiator and then were injected intravenously 6 hours later with 1×10^7^ bone marrow cells derived from the femurs of the respective donors.

### Statistical Analysis

Values represent the mean ± S.D. The statistical analysis was performed using the unpaired Studentns t-test. The statistical analysis of time-of-day-dependent variations in mRNA and protein levels was performed using a one-way analysis of variance (ANOVA). A value of P<0.05 was considered to be significant.

## Results

### Occludin and Claudin-1 Expressions Exhibit a Time of Day-dependent Variation in Colonic Epithelium, Relying on Normal Per2 Activity

**Figure 1:**
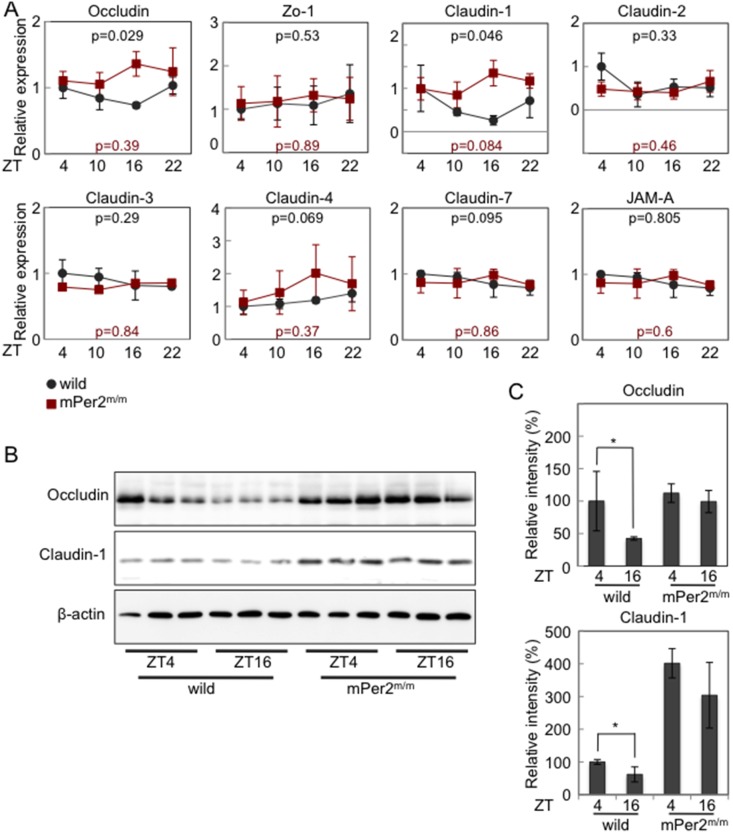
Occludin and Claudin-1 expressions exhibit a time of day-dependent variation in colonic epithelium, relying on normal Per2 activity. (**A**) Real-time PCR analysis for Occludin, Zo-1, Claudin-1, -2, -3, -4, -7, and JAM mRNAs expression in the colon tissue samples obtained from the wild-type mice and mPer2^m/m^ mice at the indicated time points (n = 8 per group). (**B**) Representative pictures of Western blot for Occludin, Claudin-1 and β-actin expression in colonic epithelial cell samples from wild-type and mPer2^m/m^ mice obtained at the indicated time points (n = 3 per group). (**C**) The quantitative analysis of (**B**) (n = 3 per group). *p<0.05.

The mRNA levels of Occludin, Zo-1, Claudin-1, -2, -3, -4, -7 and JAM-A, major components of TJ, were examined in the colon tissue samples obtained from wild-type mice and mice with the loss-of-function mutation of a key clock gene Period2 (Per2; mPer2^m/m^) every 6 hours during the day to investigate the possible circadian variations [18]. We found that the mRNA levels of Occludin and Claudin-1, but not Claudin-2, -3, -4, -7, Zo-1, and JAM-A, in the colon showed a time of day-dependent variation in wild-type mice (**Fig. 1A**). Detailed kinetic analysis of mRNA levels of Occludin and Claudin-1 at every 4 hours during the day revealed that the mRNA levels showed a nadir at Zeitgeber time (ZT) 12 (18∶00 PM) (**Fig. S1A**). We also found that Per1, Per2, Cry1, and Bmal1 mRNA levels in the colon exhibited a time of day-dependent variation (**Fig. S2**) and the kinetics of Per1 and Per2 mRNA expression were the opposite phase to Occludin and Claudin-1 mRNA kinetics (**Fig. 1A**). Consistently, the expression levels of Occludin and Claudin-1 proteins in colonic IECs showed a similar time of day-dependent variation to the mRNA expressions (**Fig. 1B and C**). Detailed kinetic analysis of protein levels of Occludin and Claudin-1 at every 4 hours during the day revealed that the protein levels showed a nadir at ZT 16 (22∶00 PM) (**Fig. S1B**), suggesting that there was a time-lag between mRNA and protein expression changes of Occludin and Claudin-1. However, please note that the circadian changes of Occludin, but not Claudin-1, protein levels did not reach a statistical significance (p = 0.053) and showed only a trend of circadian oscillations in this analysis. In contrast to wild-type mice, mPer2^m/m^ mice showed constitutively high expression levels of Occludin and Claudin-1 mRNAs and proteins without oscillations (**Fig. 1**). These results suggested that Occludin and Claudin-1 expressions in colonic epithelium showed circadian oscillations, which relied on normal Per2 activity.

### Clock and Bmal1 Bind to the E-box Element in the Promoter Regions of Occludin and Claudin-1 Genes and Affect their Trancriptional Responses

The Clock-Bmal1 heterodimer binds directly to the E-box element of the clock controlled genes (CCGs) [19], [20]. Occludin and Claudin-1 possess E-box elements in the promoter regions of the genes [21], [22]. To examine whether Clock and Bmal1 bind to the E-box elements of Occludin and Claudin-1 promoter regions, we performed CHiP assays by using IECs isolated from the colonic tissues of wild-type and mPer2^m/m^ mice every 12 hours during the day.

**Figure 2:**
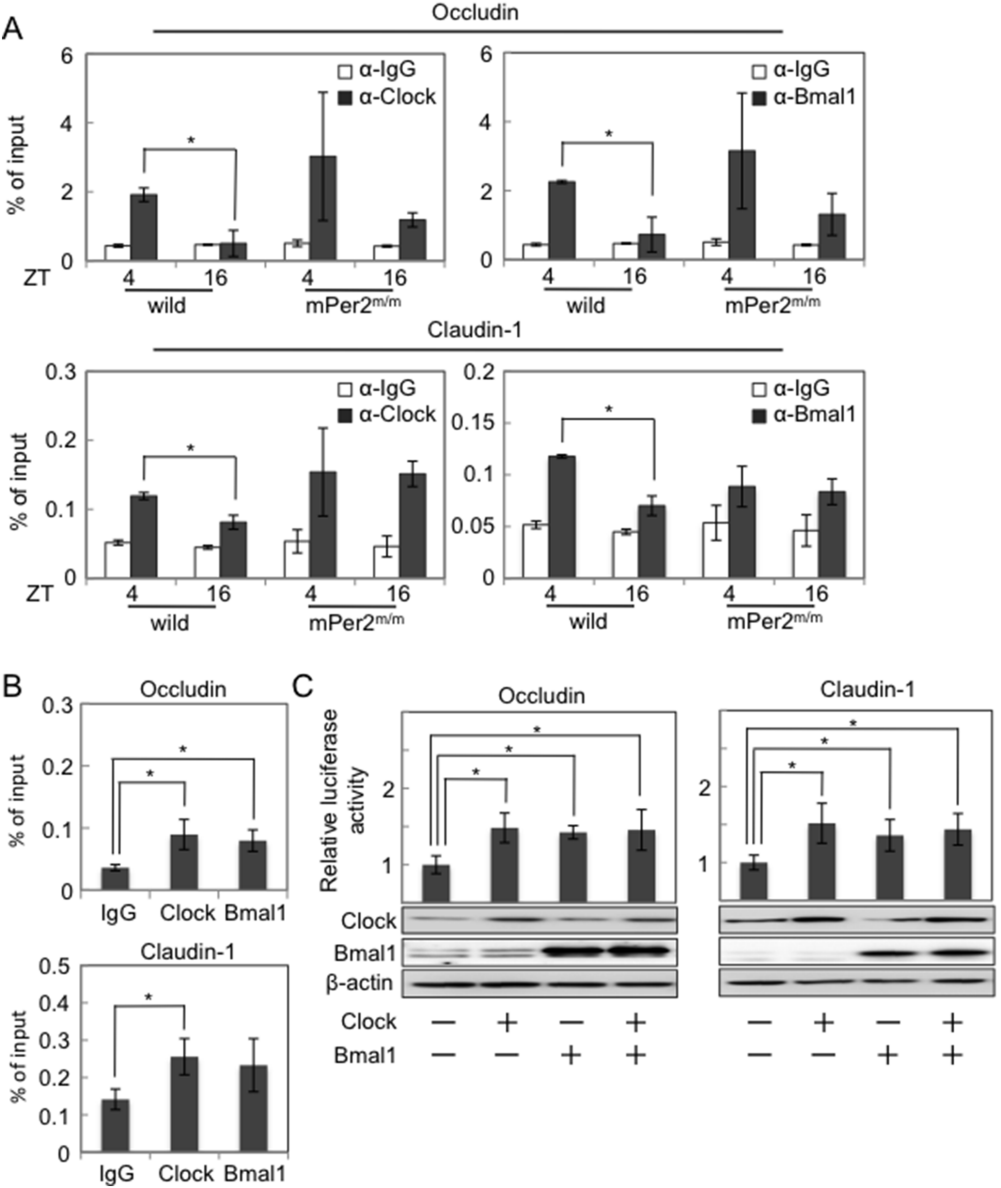
Clock and Bmal1 bind to the E-box element in the promoter regions of Occludin and Claudin-1 genes and affect their transcriptional responses. (**A**) CHiP assays for recruitments of Clock and Bmal1 to E-box elements in Occludin and Claudin-1 promoter region using colonic IECs obtained at the indicated time points from wild-type mice and mPer2^m/m^ mice (n = 3 per group). Similar results were obtained in two independent experiments. (**B**) CHiP assays for recruitments of CLOCK and BMAL1 to E-box elements in Occludin and Claudin-1 promoter region using HCT116 cells (n = 3 per group). (**C**) Relative luciferase activity in HCT116 cells transfected with Occludin and Claudin-1 promoter reporter plasmids together with Clock and Bmal1 expression vectors or a control empty vector (n = 3 per group). *p<0.05.

Clock and Bmal1 bound to the E-box elements of Occludin and Claudin-1 promoter regions in mouse colonic IECs (**Fig. 2A**). Interestingly, the ratio of binding showed a time of day-dependent variation in IECs from the colon of wild-type mice, with a high at day (ZT4: 10∶00 AM) and low at night (ZT16: 10∶00 PM) (**Fig. 2A**). However, such a variation was absent in mPer2^m/m^ mice (**Fig. 2A**). The bindings of Clock and Bmal1 to the E-box elements of Occludin and Claudin-1 promoter regions were also confirmed in human colonic epithelial cell lines HCT-116 and Caco-2 cells (**Fig. 2B** **and Fig. S3A**).

To investigate whether Clock and Bmal1 regulate transcriptional responses of Occludin and Claudin-1, we performed reporter assays using luciferase-reporter plasmids containing human Occludin (–60 to +240) or Claudin-1 (–1010 to +360) promoter fragments with E-box elements. Transfection of the Occludin- or Claudin-1 promoter reporter plasmids into HCT-116 cells showed ∼20-fold increases in luciferase activity compared with transfection of control pGL3-basic plasmid without containing the promoter elements (data not shown), suggesting that these plasmids were activated in HCT-116 cells. Overexpression of Clock or Bmal1 in HCT-116 cells further increased the luciferase activity of Occludin- or Claudin-1-reporter plasmids (**Fig. 2C**). Similar findings were also observed in Caco-2 cells (**Fig. S3B**). These results suggested that Clock and Bmal1 bound directly to the E-box elements of Occludin and Claudin-1 promoters and increased the transcriptional responses.

### Colonic Permeability is under the Circadian Control, which is Inversely Associated with Occludin and Claudin-1 Expression Levels

**Figure 3:**
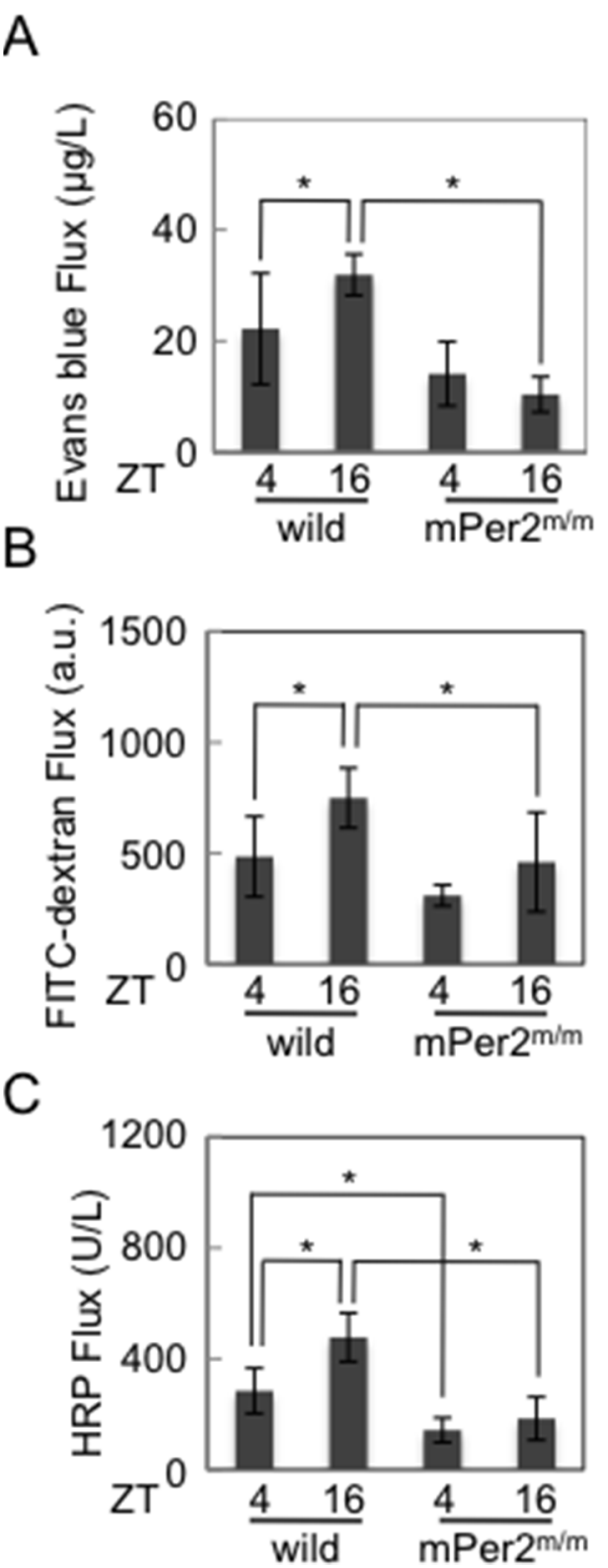
Colonic permeability is under the circadian control. (**A**) Permeability to evans blue *in vivo* at the indicated time points from wild-type mice and mPer2^m/m^ mice (n = 5 per group). (**B**) Permeability to FITC-dextran 4000 *ex vivo* obtained at the indicated time points from wild-type mice and mPer2^m/m^ mice (n = 6 per group). (**C**) Permeability to HRP *ex vivo* obtained at the indicated time points from wild-type mice and mPer2^m/m^ mice (n = 8 per group). *p<0.05.

The possibility of altered epithelial permeability in wild-type and mPer2^m/m^ mice was investigated since the TJ proteins play an important role in epithelial permeability through the control of the paracellular pathway for movement of intestinal fluid and macromolecules [1]–[3]. Colonic permeability to Evans blue *in vivo* and to FITC-dextran and HRP *ex vivo* showed a time-of-day dependent variation in wild-type mice, with a high at night (ZT16: 10∶00 PM) and low at day (ZT4: 10∶00 AM), which was the opposite phase to Occludin and Claudin-1 protein levels (**Fig. 2** **and** **3**). However, such a variation was absent in mPer2^m/m^ mice and, as expected, the colonic permeability was lower in mPer2^m/m^ mice than that in wild-type mice (**Fig. 3**). These results suggested that colonic permeability was under control of the circadian clock, which was associated with temporal alterations of colonic permeability.

### mPer2^m/m^ Mice Show Reduced Susceptibility to DSS-induced Colitis

We then investigated whether mPer2^m/m^ mice with reduced colonic permeability were more resistant to colonic injury induced by DSS, because intestinal permeability was associated with susceptibility to colitis [1]–[3], [23].

**Figure 4:**
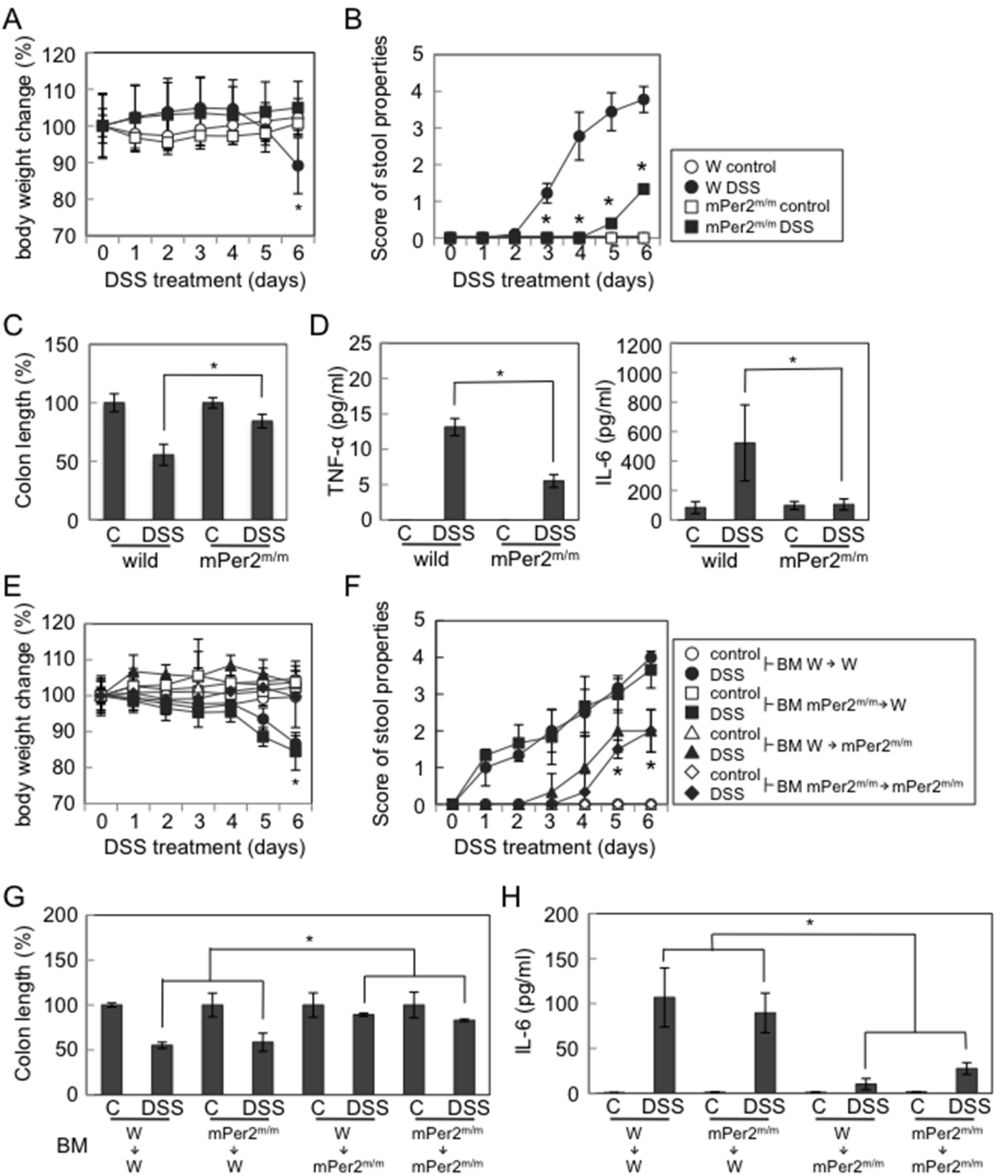
mPer2^m/m^ mice show reduced susceptibility to DSS-induced colitis. (**A**) Body weight changes in mice for 6 days after administration of 5% DSS. (**B**) Stool scores in mice for 6 days after administration of 5% DSS. (**C**) Colon length in mice on day 7 after administration of 5% DSS. (**D**) TNF-α and IL-6 levels in the colon tissue samples measured by ELISA. The samples were obtained from the mice on day 7 after administration of 5% DSS. The susceptibility to DSS-colitis in wild/mPer2^m/m^ bone marrow chimeras mice was shown (**E–H**). (**E**) Body weight changes in mice 6 days after administration of 5% DSS. (**F**) Stool scores in mice 6 days after administration of 5% DSS. (**G**) Colon length in mice on day 7 after administration of 5% DSS. (**H**) IL-6 levels measured by ELISA in the colon tissue samples obtained from the mice on day 7 after the administration of 5% DSS. n = 8 per group, *p<0.05. The mice were sacrificed at ZT4 (10∶00 AM) in Fig. **5C, D, G** and **H**.

mPer2^m/m^ mice given 5% DSS in drinking water over a period of 7 days showed less body weight loss, bloody stool, and colon shrinkage than the wild-type mice. Reduced levels of TNF-α and IL-6 proteins in the colon were observed in the mPer2^m/m^ mice in comparison to wild-type mice (**Fig. 4A–D**). The mRNA expression levels of pro-inflammatory molecular markers, myeloperoxidase (Mpo), heme oxygenase (Ho)-1, cyclooxygenase (Cox)-2, and prostaglandin E synthase (Pges)-1 in the colon were also less in mPer2^m/m^ mice than those in wild-type mice (**Fig. S4A**). mPer2^m/m^ mice drank equivalent amounts of DSS-containing water to wild-type mice per day during the period of DSS challenge (day 0–7; **Fig. S4B**). These results suggested that mPer2^m/m^ mice were more resistant to DSS-induced colitis in comparison to wild-type mice.

Bone marrow chimeric mice in which the donors and/or the recipients were either wild-type or mPer2^m/m^ mice were generated to further investigate whether the Per2 mutation in the tissue resident cells (most likely colonic epithelium) was important for the protection against DSS-induced colitis. DSS challenge of chimeric mice lacking the Per2 mutation in hematopoietic cells induced pro-inflammatory markers similar to those observed in wild-type mice reconstituted with wild-type bone marrow cells (**Fig. 4E–H** **, Fig. S5**). In contrast, chimeric mice lacking Per2 in the non-hematopoietic cells were resistant to DSS-induced colitis similar to the mPer2^m/m^ mice reconstituted with mPer2^m/m^ bone marrow cells, as indicated by the changes of those parameters (**Fig. 4E–H** **, Fig. S5**). These results suggested that the Per2 mutation conferred protection against DSS-induced colitis when it occurred in non-hematopoietic cells (presumably the cells of the colonic epithelium).

### Clock^Δ19/Δ19^ Mice Show Enhanced Susceptibility to DSS-induced Colitis

To further ascertain that the circadian clock regulated a time of day-dependent variation of Occludin and Claudin-1expression in the colon, we examined the mRNA levels of Occludin and Claudin-1 in the colon tissue samples obtained from wild-type mice and mice with the loss-of-function mutation of another key clock gene Clock (Clock**^Δ19/Δ19^** mice) every 6 hours during the day. The mRNA levels of Occludin and Claudin-1 in the colon showed a time of day-dependent variation in wild-type mice whereas the variation was absent in Clock**^Δ19/Δ19^** mice (**Fig. S6A**). Interestingly, the mRNA levels of Occludin and Claudin-1 in the colon were relative low in Clock**^Δ19/Δ19^** mice compared with those in wild-type mice. Consistently, Clock**^Δ19/Δ19^** mice were more sensitive to the colonic injury induced by DSS than wild-type mice as judged by body weight changes, stool property, and Ho-1 and Pges-1 expression (**Fig. S6B–D**). These results also suggested that disruption of the circadian clock affected TJ protein expression and barrier function. The opposite phenotypes observed in Clock**^Δ19/Δ19^** mice to those in mPer2**^m/m^** mice may be attributed to the opposite function of Clock and Per2 in the circadian clockwork; Clock acts as a positive limb whereas Per2 acts as a negative limb in the transcriptional-translational feedback loop in the molecular clock machinery.

### IL-1β and TNF-α mRNAs Expression does not Exhibit a Time of Day-dependent Variation in the Mouse Colon

Major proinflammatory cytokines IL-1β and TNF-α have been shown to regulate epithelial barrier function [2]. To investigate whether the circadian clock also affects IL-1β and TNF-α expression, we examined mRNA levels of IL-1β and TNF-α in the colon tissue samples obtained from wild-type mice and mPer2^m/m^ mice every 6 hours during the day. We found that IL-1β and TNF-α mRNA levels did not show circadian oscillations in wild-type mice and they were comparable between wild-type and mPer2^m/m^ mice (**Fig. S7**).

## Discussion

The circadian clock plays an important role in the regulation of several physiological functions of the intestine as well as other organs [7]–[9]. Most recently, Summa *et al.* also suggested that the circadian clock regulated intestinal permeability although the precise mechanisms remain largely unknown [10]. The current study demonstrated that the expressions of TJ proteins Occludin and Claudin-1 in the large intestinal epithelium showed daily oscillations, which required normal Per2 activity and the temporal changes of the expression levels were inversely associated with colonic permeability and different susceptibility to DSS-induced colitis between wild-type and mPer2^m/m^ mice. These results suggest that the circadian clock regulates a time of day-dependent variation in colonic TJ protein expression levels, thereby affecting intestinal permeability on a daily basis and also susceptibility to colitis.

One of the primary findings of this study was that 1) Occludin and Claudin-1 mRNAs showed daily oscillations, 2) Clock and Bmal1 bond directly to the E-box elements of Occludin and Claudin-1 promoters, and 3) Overexpression of Clock and Bmal1 activated Occludin and Claudin-1 promoters containing E-box elements (**Fig. 1** **and** **2**). These results suggest that Occludin and Claudin-1 are CCGs with E-box elements in the promoter region directly regulated by Clock/Bmal1 heterodimers. However, a recent study suggests that only 22% of mRNA cycling genes are driven by de novo transcription in mouse liver [24]. Thus, we can not exclude the possibility that posttranscriptional mechanisms may also underlie the circadian regulation of Occludin and Claudin-1 expression.

We showed that colonic permeability was under the circadian control, which was inversely associated with Occludin and Claudin-1 expression levels (**Fig. 3**). Claudins are defined as most important key factors for tight junction permeability and the tightness of TJ is determined by the number, composition, and the mixing ratio of Claudins [25]–[27]. Recent studies also suggest that Occludin is important for the maintenance and assembly of TJ proteins [28]–[30].

We also found that IL-1β and TNF-α mRNA levels did not show circadian oscillations in wild-type mice and they were comparable between wild-type and mPer2^m/m^ mice (**Fig. S7**). Therefore, it is likely that Clock-Bmal1 heterodimers regulate colonic permeability in a circadian manner by timing the expression level of Claudin-1 and Occludin and affect TJ function. However, it remains to be determined whether Occludin and Claudin-1 expression change are indeed primarily responsible for daily changes of tight junction permeability and susceptibility to DSS-induced colitis.

It appears that the current findings are incompatible with the previous findings by Summa et al. [10]. They reported that there were no significant differences in Occludin mRNA expression in the colon between wild-type mice and mice with a mutation of Clock (Clock^Δ19/Δ19^ mice) and there were increased cytoplasmic (i.e. decreased membrane-bound) Occludin protein levels in Clock^Δ19/Δ19^ mice compared with those in wild-type mice [10]. However, they examined Occludin mRNA and cytoplasmic Occludin protein levels in the mouse colon at only one time point in wild-type and Clock^Δ19/Δ19^ mice. They did not examine total Occludin protein levels, either. These experimental differences might explain the discrepancy between the current and previous findings. Actually, we found that the mRNA levels of Occludin and Claudin-1 in the colon showed a time of day-dependent variation in wild-type mice whereas the variation was absent in Clock**^Δ19/Δ19^** mice (**S-Fig. 6A**).

In addition, Summa et al. reported that Clock^Δ19/Δ19^ mice had increased colonic permeability compared with wild-type mice [10]. Since Clock (a positive limb of the circadian clock machinery) and Per2 (a negative limb of the circadian clock machinery) functions as an opposite direction as “clock genes”, it may be that Clock^Δ19/Δ19^ mice have opposite circadian phenotypes to mPer2^m/m^ mice. The current findings that Clock**^Δ19/Δ19^** mice were sensitive to DSS-induced colonic injury compared to wild-type mice in contrast to mPer2**^m/m^** mice (**Fig. S6**) may support this idea.

A dysfunction of TJ proteins weakens intercellular adhesion, promotes intestinal permeability, and inflammation [1], [3]. Therefore, constitutively high Occludin and Claudin-1 protein expression levels in the large intestine are likely to be attributed the reduced susceptibility to DSS-induced colitis in mPer2^m/m^ mice (**Fig. 4**). Although, it is possible that the reduced susceptibility to DSS-induced colitis in mPer2^m/m^ mice may be due to some type of immune cell dysfunction, such as NK cells and macrophages, the chimeric mouse experiments strongly suggest that the Per2 mutation in non-hematopoietic cells is responsible for the protection against DSS-induced colitis (**Fig. 4**).

It is not clear about the half-life of Occludin and Claudin-1 proteins *in vivo. In vitro* studies showed that basal half-life of Occludin was about ∼6 hours in primary human brain microvascular cells [31] and basal half-life of Claudin-1 was also about ∼6 hours in human lung carcinoma cell line A549 cells [32]. Hence, we speculate that Occludin and Claudin-1 proteins might have relatively short half-life in the mouse colon and circadian regulation of these molecules at the transcriptional levels could indeed contribute to the changes of the protein levels *in vivo*.

In summary, we suggest that expressions of TJ proteins Occludin and Claudin-1 in the large intestinal epithelium are under control of the circadian clock, which is associated with temporal changes of colonic permeability and also susceptibility to colitis. To our knowledge, this is the first study showing a novel regulation of TJs by the circadian clock. The findings would provide new insights into the regulation of intestinal barrier function and pathophysiology of colitis.

## Supporting Information

Figure S1 **Occludin and Claudin-1 expressions exhibit a time of day-dependent variation in colonic epithelium (detailed kinetic analysis).** **(A)** Real-time PCR analysis for Occludin and Claudin-1 mRNAs expression in the colonic epithelial cells samples obtained at the indicated time points from wild-type mice (n = 4 per group). **(B)** The quantitative analysis of Western blot pictures for Occludin and Claudin-1 expression in the colonic epithelial cell samples from wild-type mice obtained at the indicated time points (n = 4 per group).
(TIF)

Figure S2 **Kinetics of major “clock genes” mRNAs expression in the mouse colon.** Real-time PCR analysis for Per1, Per2, Per3, Clock, Bmal1 and Cry1 mRNAs expression in the colon tissue samples from wild-type mice and mPer2^m/m^ mice obtained at the indicated time points (n = 8 per group).
(TIF)

Figure S3 **Clock and Bmal1 bind to the E-box element in the promoter regions of Occludin and Claudin-1 genes and affect their transcriptional responses in Caco-2.** **(A)** CHiP assays for recruitments of Clock and Bmal1 to E-box elements in Occludin and Claudin-1 promoter region using Caco-2 cells (n = 3 per group). **(B)** Relative luciferase activity in Caco-2 cells transfected with Occludin and Claudin-1 promoter reporter plasmids together with Clock and Bmal1 expression vectors or a control empty vector (n = 3 per group). *p<0.05.
(TIF)

Figure S4 **mPer2^m/m^ mice show reduced susceptibility to DSS-induced colitis. (A)** Real-time PCR analysis for Mpo, Ho-1, Cox-2 and Pges1 mRNAs expression in the colon tissue obtained from the mice on day 7 after the administration of 5% DSS. **(B)** The amounts of drinking water in mice for 6 days after administration of 5% DSS. n = 8 per group, *p<0.05. The mice were sacrificed at ZT4 (10∶00 AM).
(TIF)

Figure S5 **Per2 mutation in non-hematopoietic cells is critical for protection against DSS-induced colitis.** Real-time PCR analysis for Ho-1, Cox-2 and Pges-1 mRNAs expression in the colon tissue obtained from the mice on day 7 after administration of 5% DSS. n = 8 per group, *p<0.05. The mice were sacrificed at ZT4 (10∶00 AM).
(TIF)

Figure S6 **Clock^Δ19/Δ19^ mice show enhanced susceptibility to DSS-induced colitis. (A)** Real-time PCR analysis for Occludin and Claudin-1 mRNAs expression in the colon tissue samples from wild-type mice and Clock**^Δ19/Δ19^** mice obtained at the indicated time points (n = 4 per group). **(B–D)** The severity of DSS-colitis in the wild-type and Clock**^Δ19/Δ19^** mice; Body weight changes in mice for 5 days after administration of 5% DSS (**B**). Stool scores in mice for 5 days after administration of 5% DSS (**C**). Real-time PCR analysis for Ho-1 and Pges1 mRNAs expression in the colon tissue obtained from the mice on day 6 after the administration of 5% DSS (**D**). n = 4 per group, *p<0.05. The mice were sacrificed at ZT4 (10∶00 AM).
(TIF)

Figure S7 **Kinetics of IL-1β and TNF-α mRNAs expression in the mouse colon.** Real-time PCR analysis for IL-1β and TNF-α mRNAs expression in the colon tissue samples from wild-type mice and mPer2^m/m^ mice obtained at the indicated time points (n = 4 per group).
(TIF)
