# Supplementary figures and images for: Expressions of Tight Junction Proteins Occludin and Claudin-1 Are under the Circadian Control in the Mouse Large Intestine: Implications in Intestinal Permeability and Susceptibility to Colitis
Source: PLoS One. 2014 May 20;9(5):e98016. doi: 10.1371/journal.pone.0098016 (PMC4028230; doi:10.1371/journal.pone.0098016)

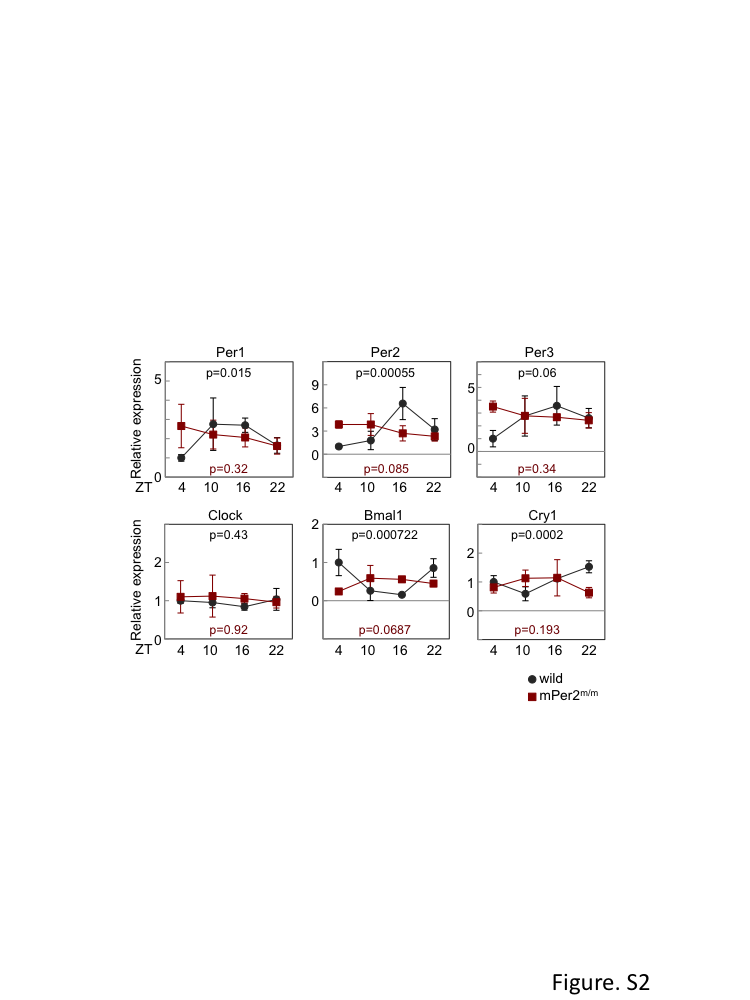

Supplement: Figure S2 — Kinetics of major “clock genes” mRNAs expression in the mouse colon. Real-time PCR analysis for Per1, Per2, Per3, Clock, Bmal1 and Cry1 mRNAs expression in the colon tissue samples from wild-type mice and mPer2m/m mice obtained at the indicated time points (n = 8 per group). (TIF) [file pone.0098016.s002.tif]

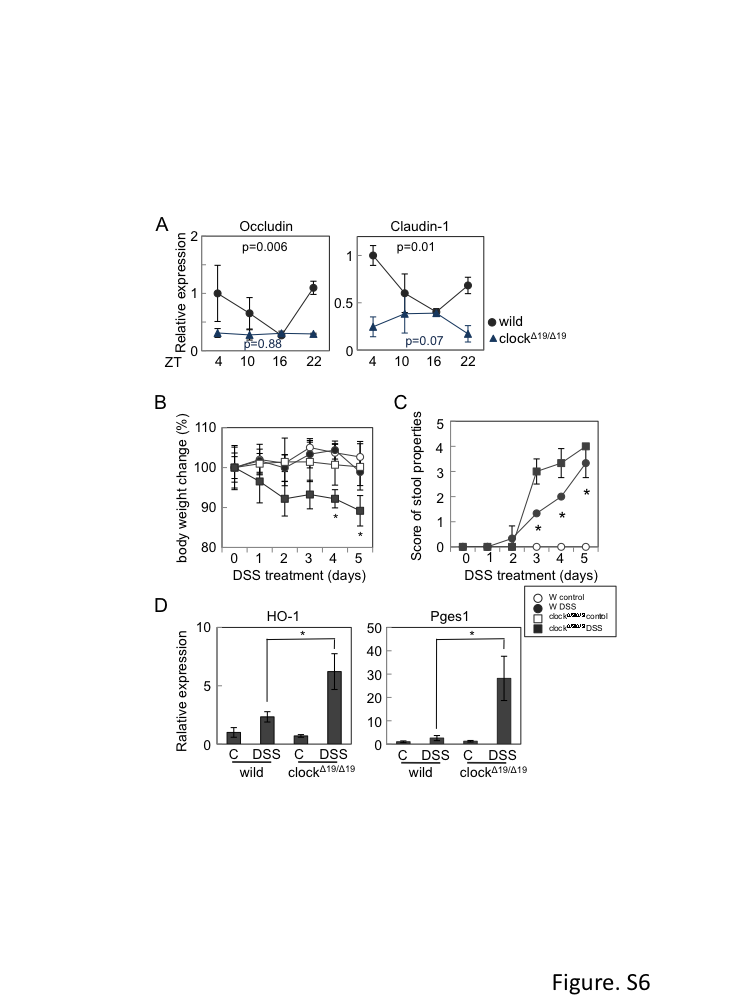

Supplement: Figure S6 — ClockΔ19/Δ19 mice show enhanced susceptibility to DSS-induced colitis. (A) Real-time PCR analysis for Occludin and Claudin-1 mRNAs expression in the colon tissue samples from wild-type mice and ClockΔ19/Δ19 mice obtained at the indicated time points (n = 4 per group). (B–D) The severity of DSS-colitis in the wild-type and ClockΔ19/Δ19 mice; Body weight changes in mice for 5 days after administration of 5% DSS (B). Stool scores in mice for 5 days after administration of 5% DSS (C). Real-time PCR analysis for Ho-1 and Pges1 mRNAs expression in the colon tissue obtained from the mice on day 6 after the administration of 5% DSS (D). n = 4 per group, *p<0.05. The mice were sacrificed at ZT4 (10∶00 AM). (TIF) [file pone.0098016.s006.tif]

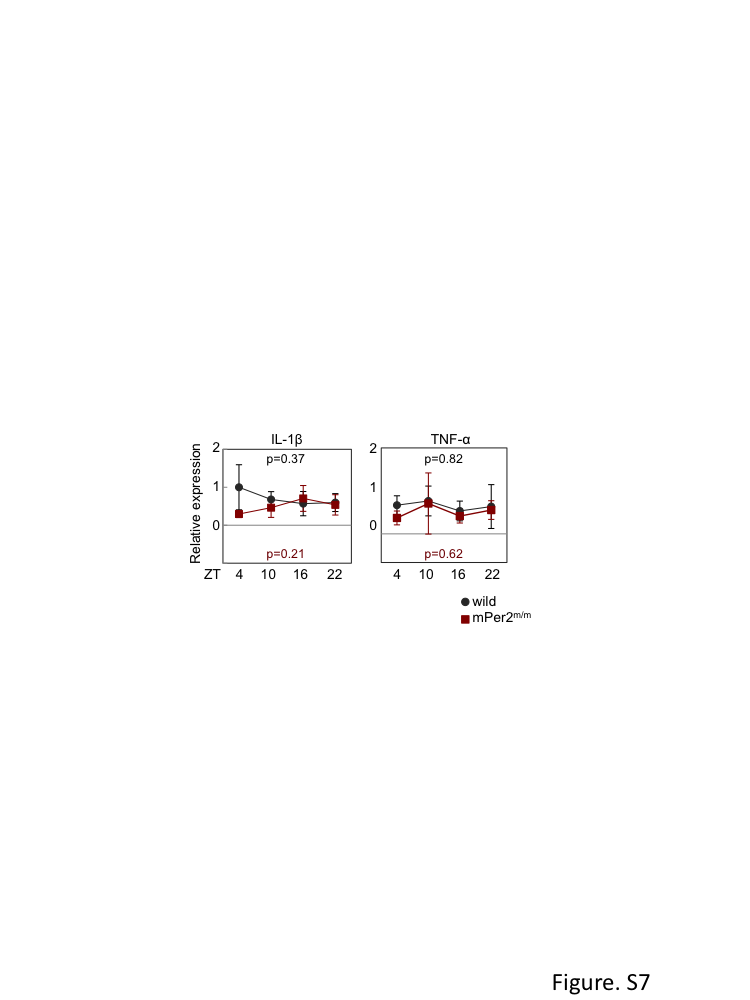

Supplement: Figure S7 — Kinetics of IL-1β and TNF-α mRNAs expression in the mouse colon. Real-time PCR analysis for IL-1β and TNF-α mRNAs expression in the colon tissue samples from wild-type mice and mPer2m/m mice obtained at the indicated time points (n = 4 per group). (TIF) [file pone.0098016.s007.tif]
